# Caveolin-1 Modulates Islet Amyloid Polypeptide Expression Through Interaction with TXNIP in Murine Pancreatic β-Cells

**DOI:** 10.3390/biomedicines14061344

**Published:** 2026-06-15

**Authors:** Kunying Liu, Xubin Yang, Shuo Lin, Chuwen Lin, Nan Cai, Longyi Zeng, Wen Zeng

**Affiliations:** 1 Department of Endocrinology & Metabolism, The Third Affiliated Hospital of Sun Yat-sen University, Guangzhou 510630, China; liukunying@yahoo.com (K.L.); yxbin@mail.sysu.edu.cn (X.Y.); gzlinshuo@hotmail.com (S.L.); linchuwenn@163.com (C.L.); cain5@sysu.com (N.C.); 2 Guangdong Provincial Key Laboratory of Diabetology, The Third Affiliated Hospital of Sun Yat-sen University, Guangzhou 510630, China; 3 Guangzhou Municipal Key Laboratory of Mechanistic and Translational Obesity Research, The Third Affiliated Hospital of Sun Yat-sen University, Guangzhou 510630, China; 4 Medical Center for Comprehensive Weight Control, The Third Affiliated Hospital of Sun Yat-sen University, Guangzhou 510630, China

**Keywords:** caveolin-1, islet amyloid polypeptide, pancreatic β cells, thioredoxin-interacting protein

## Abstract

**Background**: Pathological aggregation of islet amyloid polypeptide (IAPP) contributes to β-cell dysfunction in type 2 diabetes. Our previous studies demonstrated that caveolin-1 (Cav-1) deficiency protects β-cells from palmitate-induced apoptosis. Microarray profiling further indicated that Cav-1 silencing alters IAPP expression. This study aimed to investigate the effects of Cav-1 depletion on IAPP secretion and expression and to explore the potential involvement of thioredoxin-interacting protein (TXNIP). **Methods**: We performed lentiviral-mediated Cav-1 knockdown in NIT-1 cells and isolated murine islets, and simultaneously generated an inducible β-cell-specific Cav-1 knockout (iβ-Cav1 KO) mouse model. IAPP secretion and expression were assessed by ELISA, Western blot, qPCR and immunofluorescence. The expression of IAPP-processing enzymes (PAM, PC1, and PC2) and degradation factors (IDE and BACE2) was examined. Co-immunoprecipitation (Co-IP) and immunofluorescence were performed to investigate the interaction between Cav-1 and TXNIP. **Results**: Cav-1 depletion significantly reduced both IAPP secretion and expression in vitro and in vivo. High-fat-diet-fed iβ-Cav1 KO mice exhibited the lowest serum IAPP levels. Mechanistically, Cav-1 depletion was associated with downregulation of PAM, PC1, and PC2 and upregulation of IDE and BACE2. Additionally, Cav-1 depletion decreased TXNIP expression. Immunofluorescence revealed co-localization of Cav-1 and TXNIP, and co-immunoprecipitation further demonstrated their direct physical interaction. **Conclusions**: Cav-1 is essential for IAPP secretion and expression in β-cells. The direct physical interaction between Cav-1 and TXNIP suggests that TXNIP may mediate the regulatory effects of Cav-1 on IAPP processing or secretion. These findings identify the Cav-1–TXNIP axis as a potential target for mitigating IAPP-related β-cell dysfunction.

## 1. Introduction

The global surge in type 2 diabetes mellitus (T2DM) prevalence—driven by population aging and caloric excess—has made it one of the most pressing metabolic pandemics of the 21st century [1,2]. The core pathophysiology centers on a progressive failure of pancreatic islet β-cells to release sufficient insulin. However, the molecular events precipitating β-cell decompensation remain incompletely defined [3]. Over the past three decades, autopsy series and prospective clinical cohorts have consistently documented islet amyloid deposition in >90% of patients with T2DM. The abundance of these fibrillar aggregates—formed by misfolded islet amyloid polypeptide (IAPP)—correlates tightly with β-cell loss and disease severity [4,5]. Transgenic rodents expressing human IAPP spontaneously develop islet amyloid, triggering inflammation, endoplasmic-reticulum stress, and apoptosis. These findings establish that an imbalance in IAPP synthesis–folding–clearance is a critical driver of β-cell demise [6,7]. Consequently, suppressing IAPP production and aggregation is considered a plausible therapeutic strategy for T2DM.

The membrane microdomain scaffolding protein caveolin-1 (Cav-1) has emerged as a key regulator of glucose and lipid homeostasis [8]. Cav-1 is highly enriched in β-cells, where its scaffolding domain interacts with the insulin receptor, GLUT2, and multiple vesicular trafficking proteins to fine-tune insulin secretion and signal transduction [9,10]. Our previous work demonstrated that Cav-1 silencing in NIT-1 cells and isolated murine islets protects against palmitate-induced lipotoxicity, as evidenced by enhanced glucose-stimulated insulin secretion (GSIS), reduced apoptosis, and increased cell viability [11,12,13]. Mechanistically, Cav-1 depletion attenuated endoplasmic reticulum stress and attenuated inflammation [11,12]. In a subsequent study using the inducible β-cell-specific Cav-1 knockout (iβ-Cav1 KO) mouse model, we found that Cav-1 depletion prevented high-fat-diet-induced hyperglycemia and insulin resistance and improved glucose tolerance in vivo [12,13]. Furthermore, our genome-wide microarray profiling of primary mouse islets with Cav-1 knockdown revealed a reduction in IAPP mRNA expression, suggesting a potential role for Cav-1 in regulating IAPP proteostasis. However, whether Cav-1 directly governs IAPP proteostasis—that is, the synthesis, folding, and clearance of IAPP—and thereby determines β-cell fate remains unknown.

Based on these findings, we hypothesize that Cav-1 governs β-cell mass and function by regulating IAPP proteostasis. Attenuation of Cav-1 signaling would suppress pathogenic IAPP accumulation by dampening its biosynthesis and/or facilitating its proteolytic clearance, thereby shielding β-cells from amyloid-induced cytotoxicity and attenuating T2DM progression. While the functional outcomes of Cav-1 modulation have been characterized in our previous studies [11,12,13,14], the upstream molecular mechanisms linking Cav-1 to IAPP regulation remain unknown. In this study, we employed lentiviral-mediated Cav-1 knockdown in NIT-1 cells and primary islets and generated iβ-Cav1 KO mice to investigate the effect of Cav-1 depletion on IAPP synthesis and degradation, thereby elucidating the mechanistic basis for the previously observed functional improvements.

## 2. Materials and Methods

### 2.1. Generation of Cav-1 Knockdown Insulinoma Cell and Pancreatic Islets

The NIT-1 mouse insulinoma cell line was procured from the American Type Culture Collection (ATCC, Manassas, VA, USA) and maintained in low-glucose DMEM medium enriched with 10% fetal bovine serum. Pancreatic islets were isolated from 8- to 12-week-old mice obtained from the Guangzhou Medical Laboratory Animal Center (Guangzhou, China). For lentiviral transduction, shRNA sequences targeting Cav-1 (Cav1-shRNA: 5′-ACGTGGTCAAGATTGACTT-3′) or a scrambled control sequence (Ctrl-shRNA: 5′-TTCTCCGAACGTGTCACGT-3′) were cloned into the EGFP-labeled lentiviral vector GV248 (GENECHEM, Shanghai, China). NIT-1 cells and freshly isolated mouse islets were transduced at a multiplicity of infection (MOI) of 20 and 100, respectively, for 12 h (NIT-1) or 24 h (islets). The transduction mixture was then replaced with regular culture medium, and cells were cultured for an additional 72 h. Transduction efficiency was visually monitored by EGFP fluorescence, and successful Cav-1 depletion was validated by quantitative PCR (qPCR) and immunoblotting or immunofluorescence, as previously described and validated in our published study [11]. Cells and islets were subsequently harvested for simultaneous collection of mRNA and protein samples for subsequent analysis.

### 2.2. Generation and Housing of iβ-Cav1 KO Mice

All animal procedures were conducted in accordance with the guidelines approved by the Institute Animal Care and Use Committee (IACUC) at the Animal Ethics Committees of Sun Yat-Sen University (Approval number: SYSU-IACUC-2020-000216, Approval date: 29 June 2020). To generate iβ-Cav1 KO mice, we crossed Cav-1+flox/flox conditional knockout mice (obtained from Cyagen Biosciences, Suzhou, China) with Cre mice (genotype: B6.Cg-Tg(Ins1-cre/ERT)1Lphi/J; Jackson Laboratory, Sacramento, CA, USA). The resulting Cav-1+flox/flox; Cre+ mice were administered tamoxifen (MedChemExpress Company; Monmouth Junction, NJ, USA) (i.p., 75 mg/kg body weight for 7 consecutive days) to induce Cre-mediated recombination and achieve β-Cav-1 deletion as previously described [14]. Eight-week-old flox/flox male mice that received tamoxifen were designated as the wild-type (WT) group. The iβ-Cav1 KO mice model used in this study was from the same batch as previously validated [13,14]. Knockout efficiency in islet β-cells was confirmed by immunofluorescence [14]. For all subsequent analyses of IAPP synthesis and degradation enzymes, we compared the WT+HFD (wild-type + high-fat diet) and KO+HFD (knockout before high-fat diet) groups to directly assess the effect of Cav-1 deletion under high-fat-diet conditions. Subsequently, all groups, including the WT, and KO groups, were fed either a control diet (CD) (WT+CD group, KO+CD group) or a high-fat diet (HFD) (WT+HFD group, KO+HFD group). A detailed flow chart of the in vivo experimental design is provided in Supplementary Figure S1 [12].

Animals were housed under a standard 12:12 h light–dark cycle at a constant temperature of 21 °C. The mice had free access to either a commercial control diet (CD) (4% fat (wt/wt), Guangdong Medical Laboratory Animal Center) or a HFD (60% fat, D12492, Research Diets, New Brunswick, NJ, USA) and water for 16 weeks.

### 2.3. ELISA

Murine serum and pancreatic islets were collected and stored at –80 °C for subsequent Enzyme-Linked Immunosorbent Assay (ELISA) analysis. The Mouse Islet Amyloid Polypeptide ELISA (Invitrogen, Carlsbad, CA, USA) protocol was followed precisely. The standard was serially diluted using 1× assay buffer to create a concentration gradient. Meanwhile, the primary antibody and biotinylated peptide were reconstituted in 5 mL of 1× assay buffer. Subsequently, 50 µL of each sample and 50 µL of each standard were aliquoted into their respective wells, followed by the addition of 50 µL of the primary antibody to both sample and standard wells. The plates were then incubated at room temperature, with gentle shaking, for 2 h. After the incubation period, the solution was removed, and 100 µL of SA-HRP secondary antibody was added to each well, followed by another incubation at room temperature for 1 h. Upon completion of this step, 100 µL of TMB substrate was added to each well and incubated for 30 min. Finally, 100 µL of stop solution (HCl) was added to terminate color development. Absorbance was measured at 450 nm using a microplate reader (Thermo Fisher Scientific, Waltham, MA, USA).

### 2.4. Western Blotting

Cells were washed three times with cold PBS and subsequently homogenized using cell lysis buffer supplemented with protease and phosphatase inhibitors. Western blot analysis of the cell lysates was performed as described previously [zw2018]. The primary antibodies used in this study were Caveolin-1 (Cat. #3267,1:1000), Prohormone Convertase-1 (PC1, Cat. #18030,1:1000), Prohormone Convertase-2 (PC2, Cat. #14013,1:1000), TXNIP (Cat. #14715,1:1000), β-actin (Cat. #8457,1:1000), and GAPDH (Cat. #5174,1:1000), all purchased from Cell Signaling Technology (Danvers, MA, USA). IAPP (Cat. #NBP1-06579,1:800) and PAM (Cat. #NBP2-34075,1:1000) were obtained from Novus Biologicals; BACE2 (Cat. #ab270458,1:1000) was obtained from Abcam (Cambridge, UK); and IDE (Cat. #DF6515,1:1000) was obtained from Affinity Biosciences (Cincinnati, OH, USA). All primary antibodies were diluted according to the manufacturers’ instructions. Following 12 h incubation with primary antibodies at 4 °C, the membranes were further incubated with DyLight 800-conjugated secondary antibodies (1:10,000 dilution, Thermo Fisher Scientific) for 1 h at room temperature, protected from light. Finally, the signals were visualized using the Odyssey Infrared Imaging system (LI-COR Biosciences, Lincoln, NE, USA). Protein levels were quantified and normalized to β-actin and GAPDH as internal controls using ImageJ v1.52a software (National Institutes of Health, Bethesda, MD, USA).

### 2.5. QPCR

Total RNA was isolated from cells using TRIzol reagent (Sigma-Aldrich, St. Louis, MO, USA)and subsequently reverse-transcribed into cDNA using the PrimeScript^TM^ RT Reagent Kit (Cat. #RR036A, Takara Bio, Shiga, Japan). Quantitative real-time Polymerase Chain Reaction (qPCR) was performed using the SYBR^@^ Premix Ex Taq ^TM^ II kit (Cat. #RR038, Takara Bio Inc., Shiga, Japan) on a LightCycler^@^ 480II Real-Time PCR system (Roche Diagnostics, Mannheim, Germany), with β-actin employed as an endogenous control for normalization. Relative gene expression levels were determined using the comparative Ct method (also known as the 2^(−ΔΔCt) method). The specific primer sequences used for qPCR are provided in Supplementary Table S1.

### 2.6. Immunofluorescence Analysis

For immunofluorescence analysis, frozen pancreatic tissues were sectioned into 10 μm cross-sections, and NIT-1 cells were fixed with 4% paraformaldehyde for 30 min, followed by three washes with phosphate-buffered saline (PBS) for 5 min each. The fixed cells were permeabilized with 0.1% Triton X-100 for 10 min, while pancreatic tissues were permeabilized with 0.3% Triton X-100 for 2 h. Subsequently, the samples were blocked with 5% Bovine Serum Albumin (BSA) in PBS for 1 h at room temperature and washed three times with PBS for 5 min each. Primary antibodies were applied, including Caveolin-1 (Cat. #3267, mouse anti-caveolin-1, Cell Signaling Technology, dilution 1:200), Insulin (Cat. #ab7842, guinea pig anti-insulin, Abcam, dilution 1:50), PAM (Cat. #DF8228, mouse anti-PAM, Affinity, dilution 1:50), PC1 (Cat. #ab220363, mouse anti-PC1, Abcam, dilution 1:100), PC2 (Cat. #14013S, mouse anti-PC2, Cell Signaling Technology, dilution 1:100), BACE2 (Cat. #ab270458, mouse anti-BACE2, Abcam, dilution 1:100), IDE (Cat. #DF6515, mouse anti-IDE, Affinity, dilution 1:100), and TXNIP (Cat. #ab232330, mouse anti-TXNIP, Abcam, dilution 1:100). Samples were then incubated overnight at 4 °C. After incubation, the coverslips were washed with PBS and incubated with appropriate species-specific secondary antibodies for 1 h at room temperature. Cell nuclei were stained with DAPI (1 μg/mL, Roche) for 5 min, followed by washing with PBS. Slides were then mounted using Prolong Gold Antifade Mountant (Life Technologies, Carlsbad, CA, USA). Fluorescence images were captured using a fluorescence microscope (Nikon Eclipse Ti, Tokyo, Japan), and fluorescence intensity was quantified using ImageJ software (National Institutes of Health, USA). Islets of comparable size were selected for analysis across all groups. For each islet, multiple insulin-positive β-cell regions were outlined as regions of interest (ROIs), and the mean gray value of the red channel was measured within each ROI. Values from all ROIs within the same islet were averaged to obtain a single mean fluorescence intensity value per islet [15]. Data are presented as mean fluorescence intensity (arbitrary units, a.u.).

### 2.7. Co-Immunoprecipitation

Co-immunoprecipitation (Co-IP) assays were performed to detect the interaction between Caveolin-1 (Cav-1) and TXNIP in NIT-1 cells. Briefly, cells were lysed in ice-cold RIPA lysis buffer (50 mM Tris-HCl, pH 7.4, 150 mM NaCl, 1% NP-40, 0.5% sodium deoxycholate, 0.1% SDS) supplemented with protease inhibitor cocktail and phosphatase inhibitors. After centrifugation at 13,000× *g* for 10 min at 4 °C, the supernatant was collected as total cell lysate.

For immunoprecipitation, 500 μg of protein lysate were incubated with 4 μg of anti-Cav-1 antibody or anti-TXNIP antibody, or with equal amounts of normal rabbit IgG (as a negative control), overnight at 4 °C with gentle rotation. Protein A/G magnetic beads (0.25 mg) were then added, and the mixture was incubated for 1 h at 4 °C. The beads were washed five times with ice-cold lysis buffer to remove non-specifically bound proteins. The immunoprecipitated complexes were eluted by boiling in 2× SDS loading buffer, resolved by SDS-PAGE, and analyzed by Western blotting using anti-TXNIP (Cat. #14715, Cell Signaling Technology, dilution 1:1000) and anti-Cav-1 antibodies Caveolin-1 (Cat. #3267, Cell Signaling Technology, dilution 1:1000), respectively. The input lanes represent 10% of the total cell lysate used in each immunoprecipitation reaction.

### 2.8. Statistical Analysis

Statistical analysis was performed using SPSS 21.0 software, with quantitative data expressed as mean ± standard deviation (Mean ± SD). Differences between groups were assessed using *t*-tests or one-way analysis of variance followed by appropriate post hoc tests. A *p*-value of less than 0.05 was considered statistically significant. Data visualization and further analyses, such as curve fitting and regression analysis, were conducted using GraphPad Prism 8 software (Graph Pad Software, San Diego, CA, USA).

## 3. Results

### 3.1. Cav-1 Deficiency Decreased IAPP in Murine Pancreatic Islets and in NIT-1 Cells

**Figure 1:**
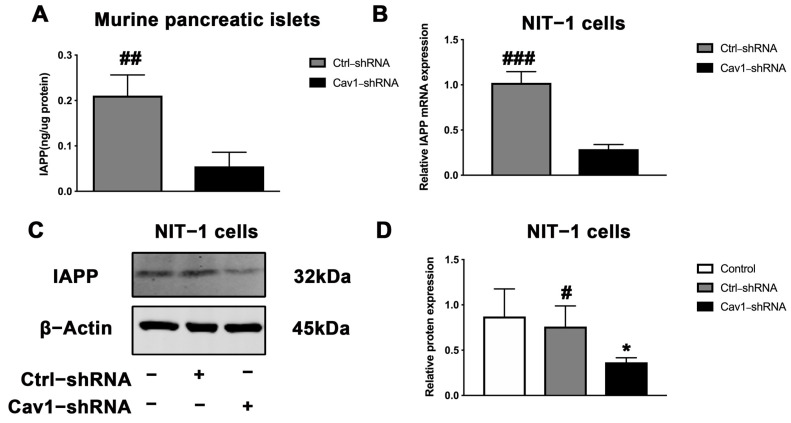
Expression of IAPP in stably transfected NIT-1 cells and isolated mouse islets. (**A**): IAPP in the supernatant of murine pancreatic islets; (**B**): IAPP mRNA was measured by qPCR in NIT-1 cells; (**C**,**D**): IAPP protein in NIT-1 cells were determined by Western blot and standardized to expression of β-Actin in NIT-1 cells; data are representative of three independent experiments (n = 3, mean ± SD). * = *p* < 0.05 between Cav1-shRNA group and Control group; ^#^ = *p* < 0.05, ^##^ = *p* < 0.01 and ^###^ = *p* < 0.001 between Cav1-shRNA group and Ctrl-shRNA group.

We first assessed the effect of Caveolin-1 silencing in isolated murine pancreatic islets using lentivirus-mediated knockdown. Compared to the control shRNA group, Caveolin-1 silencing significantly reduced IAPP secretion (0.06 ± 0.03 ng/mL vs. 0.21 ± 0.05 ng/mL, *p* < 0.01) (Figure 1A). Subsequently, we evaluated the impact of Caveolin-1 knockdown in NIT-1 cells. Consistent with the islet data, silencing Caveolin-1 led to a significant decrease in IAPP mRNA levels (0.29 ± 0.05 vs. 1.02 ± 0.12, *p* < 0.001) (Figure 1B). Additionally, Caveolin-1 knockdown significantly reduced IAPP protein expression (0.37 ± 0.05 in knockdown vs. 0.87 ± 0.30 in control, *p* < 0.05) (Figure 1C,D).

### 3.2. Cav-1 Deficiency Down-Regulated IAPP Synthesis-Related Enzymes and Up-Regulated Degradation-Related Enzymes in NIT-1 Cells

**Figure 2:**
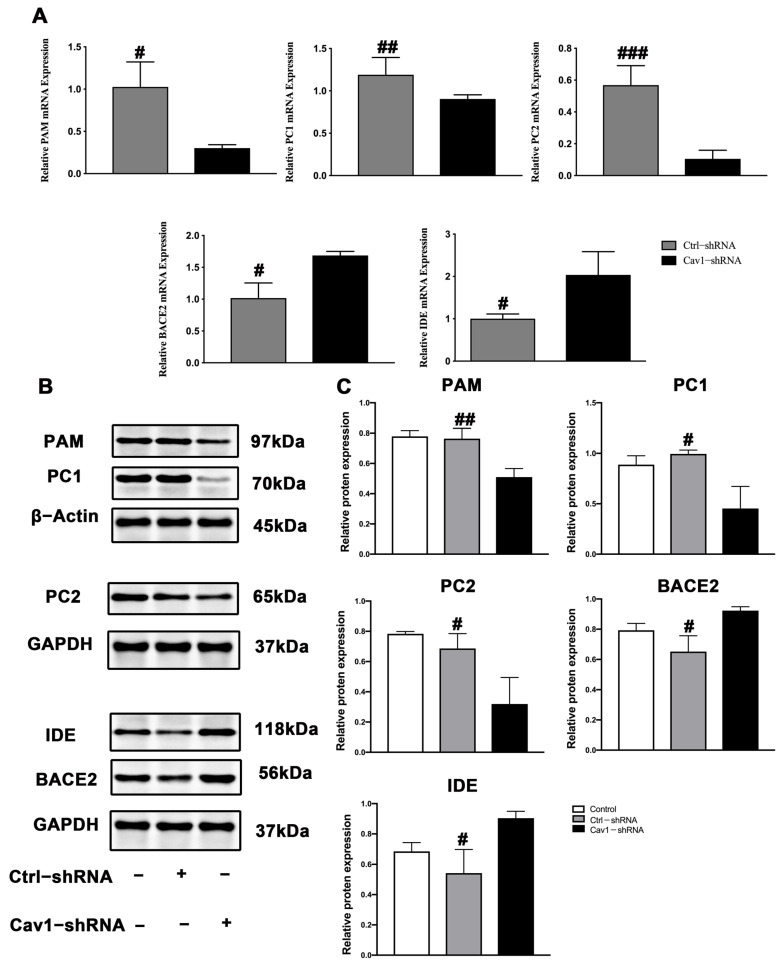
Expression of enzymes related with IAPP synthesis and degradation in stably transfected NIT-1 cells. (**A**): PAM, PC1, PC2, BACE2, and IDE mRNA expression was measured by qPCR in NIT-1 cells. (**B**,**C**): Protein content of PAM, PC1, PC2, BACE2, and IDE was determined by Western blot and standardized to expression of β-Actin or GAPDH in NIT-1 cells. Data are representative of three independent experiments (n = 3, mean ± SD. ^#^ = *p* < 0.05, ^##^ = *p* < 0.01 and ^###^ = *p* < 0.001 between Cav1-shRNA group and Ctrl-shRNA group.

To clarify how Cav-1 governs IAPP production, we quantified the enzymes involved in IAPP synthesis and degradation in NIT-1 cells after Cav-1 silencing. Relative to scrambled shRNA controls, lentiviral Cav-1 silencing lowered mRNA levels of the three IAPP-synthesizing enzymes: PAM (0.30 ± 0.04 vs. 1.03 ± 0.30, *p* < 0.05), PC1 (0.90 ± 0.05 vs. 1.19 ± 0.20, *p* < 0.01), and PC2 (0.11 ± 0.05 vs. 0.57 ± 0.12, *p* < 0.001). qPCR revealed that IDE and BACE2 transcript levels were 2.03 ± 0.55-fold and 1.66 ± 0.06-fold higher, respectively, in Cav-1-silenced NIT-1 cells than in scrambled shRNA controls (both *p* < 0.05) (Figure 2A). Consistently, Western blot analysis confirmed the transcriptional data, showing decreased protein abundance of PAM (0.51 ± 0.06 vs. 0.76 ± 0.07, *p* < 0.01), PC1 (0.45 ± 0.22 vs. 0.99 ± 0.04, *p* < 0.05), and PC2 (0.32 ± 0.18 vs. 0.69 ± 0.10, *p* < 0.05) and increased protein abundance of IDE (0.90 ± 0.04 vs.0.54 ± 0.15, *p* < 0.05) and BACE2 (0.92 ± 0.03 vs. 0.65 ± 0.10, *p* < 0.05) in Cav-1-silenced NIT-1 cells compared to scrambled shRNA controls (Figure 2B,C). These data indicated that Cav-1 silencing impaired IAPP synthesis and accelerated IAPP clearance.

### 3.3. Cav-1 Deficiency Lowers TXNIP Expression in NIT-1 Cells

**Figure 3:**
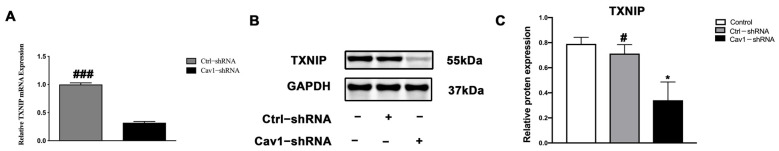
Expression of enzyme TXNIP in stably transfected NIT-1 cells. (**A**): TXNIP mRNA expression was measured by qPCR in NIT-1 cells, (**B**,**C**): Protein content of TXNIP was determined by Western blot and standardized to expression of GAPDH in NIT-1 cells. Data are representative of three independent experiments (n = 3, mean ± SD). * = *p* < 0.05 between Cav1-shRNA group and Control group; ^#^ = *p* < 0.05, ^###^ = *p* < 0.001 between Cav1-shRNA group and Ctrl-shRNA group.

To explore the potential mechanism linking Cav-1 to IAPP regulation, we examined TXNIP, an oxidative stress sensor previously identified as a positive regulator of IAPP transcription. Following lentiviral Cav-1 knockdown, TXNIP mRNA levels decreased to 0.32 ± 0.02 times those of the scrambled shRNA control (0.32 ± 0.02 vs.1.00 ± 0.03; *p* < 0.001) (Figure 3A). Consistently, Western blot analysis revealed that TXNIP levels were reduced compared to GAPDH (Cav-1 knockdown: 0.34 ± 0.15 vs. scrambled shRNA: 0.71 ± 0.07, *p* < 0.05) (Figure 3B,C).

### 3.4. Cav-1 Deletion Lowers IAPP of the Serum and Islet in iβ-Cav1 KO Mice

**Figure 4:**
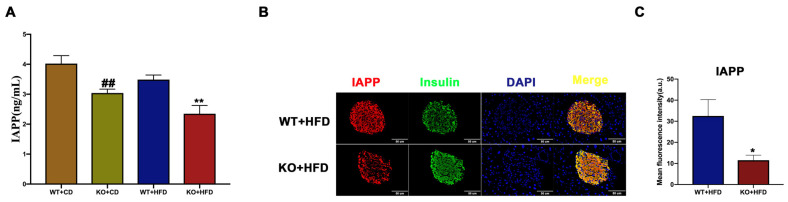
Expression of islet amyloid polypeptide in iβ-Cav1 KO (knock out) mice. (**A**): Secretion of IAPP in the serum of the mice in WT+CD (wild- type + control diet), KO+CD (knockout + control diet), WT+HFD (wild-type + high-fat diet) and KO+HFD (knockout before high-fat diet) groups. Statistical analyses were performed between genotype-matched groups under the same dietary condition (WT+CD vs. KO+CD; WT+HFD vs. KO+HFD). No significant differences were observed between diet-matched wild-type groups (WT+CD vs. WT+HFD, *p* > 0.05); (**B**,**C**): Representative fluorescent microscope images (**B**) and quantification of mean fluorescence intensity (**C**) of IAPP (red) and insulin (green) staining in islets from WT+HFD and KO+HFD mice only. Nuclei were counterstained with DAPI (blue). Data here are representative of three independent experiments (n = 3, mean ± SD). ^##^ = *p* < 0.01 between KO+CD group and WT+CD group; * = *p* < 0.05, ** = *p* < 0.01 between KO+HFD group and WT+HFD group. Images of the islets were taken using a microscope under 400× objective lens. Scale bar, 50 μm.

To extend the in vitro findings to an intact organism, we quantified IAPP secretion in iβ-Cav-1 knockout (KO) mice. Animals subjected to a HFD for 16 weeks after Cav-1 knockout (KO+HFD) exhibited 33% lower serum IAPP concentrations than wild-type littermates on the same diet (WT+HFD vs. KO+HFD, 3.49 ± 0.15 vs. 2.35 ± 0.27 ng/mL, *p* < 0.01) (Figure 4A). Under the chow diet, iβ-Cav-1 knockout mice also displayed a 24% decrease in circulating IAPP relative to WT controls (WT+CD vs. KO+CD, 4.02 ± 0.27 ng/mL vs. 3.04 ± 0.13 ng/mL, *p* < 0.01). In islets from iβ-Cav-1 KO mice subjected to the KO+HFD sequence, immunofluorescence revealed a marked reduction in IAPP synthesis, with mean fluorescence intensity per islet decreasing by 65% (WT+HFD vs. KO+HFD, 32.52 ± 7.77 vs. 11.54 ± 2.40, *p* < 0.05) (Figure 4B,C), while insulin staining remained comparable between genotypes.

### 3.5. Cav-1 Depletion Down-Regulated IAPP Synthesis-Related Enzymes in iβ-Cav1 KO Mice

**Figure 5:**
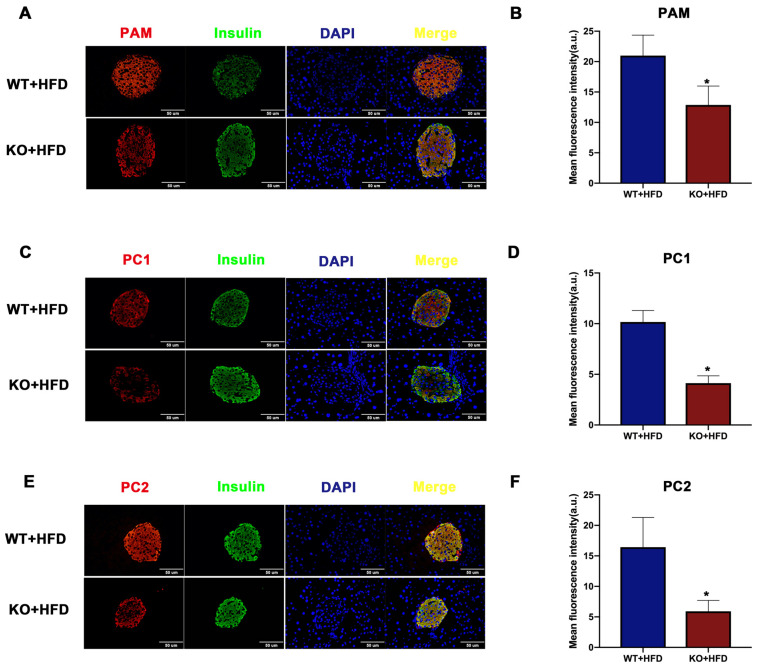
Expression of enzyme related to IAPP synthesis in the islets of the WT+HFD and KO+HFD mice. (**A**–**F**): Representative fluorescent microscope images (**A**,**C**,**E**) and quantification of mean fluorescence intensity (**B**,**D**,**F**) of PAM, PC1, and PC2 (red) and insulin (green) staining in islets from WT+HFD and KO+HFD mice. Nuclei were counterstained with DAPI (blue); Data here are representative of three independent experiments (n = 3, mean ± SD). * = *p* < 0.05 between KO+HFD group and WT+HFD group. Images of the islets were taken using a microscope under 400× objective lens. Scale bar, 50 μm.

The in vivo findings recapitulated the cellular data. In islets from iβ-Cav1 KO mice maintained on a HFD, immunofluorescence revealed a marked reduction in the IAPP-synthetic machinery, with mean fluorescence intensity per islet falling by 39% for PAM (12.87 ± 3.12 vs. 20.98 ± 3.37, *p* < 0.05), 59% for PC1 (4.13 ± 0.12 vs. 10.17 ± 1.13, *p* < 0.05), and 64% for PC2 (5.91 ± 1.79 vs. 16.43 ± 4.87, *p* < 0.05) relative to WT+HFD controls, whereas the insulin signal remained unchanged (Figure 5A–F).

### 3.6. Cav-1 Depletion Up-Regulated IAPP Degradation-Related Enzymes in iβ-Cav1 KO Mice

**Figure 6:**
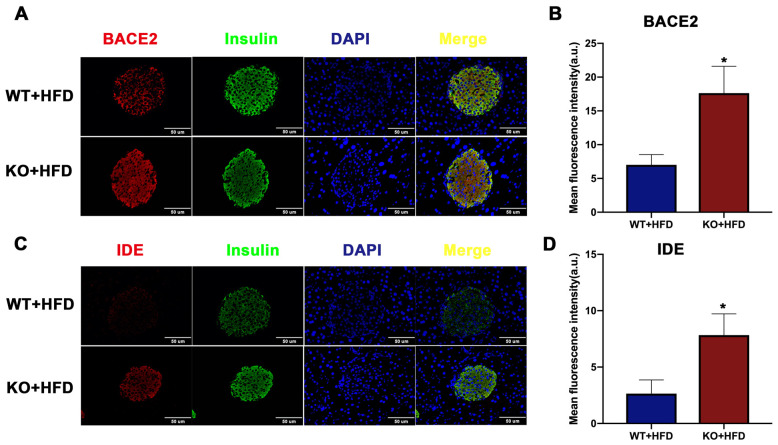
Expression of enzyme related to IAPP degradation in the islet of the WT+HFD and KO+HFD mice. (**A**–**D**): Representative fluorescent microscope images (**A**,**C**) and quantification of mean fluorescence intensity (**B**,**D**) of BACE2 and IDE (red) and insulin (green) staining in islets from WT+HFD and KO+HFD mice. Nuclei were counterstained with DAPI (blue); Data here are representative of three independent experiments (n = 3, mean ± SD). * = *p* < 0.05 between KO+HFD group and WT+HFD group. Images of the islets were taken using a microscope under 400× objective lens. Scale bar, 50 μm.

Consistent with the in vitro observations, Cav-1 deletion in HFD mice significantly elevated the islet expression of IAPP-degrading enzymes. Immunofluorescence analysis demonstrated that mean fluorescence intensity per islet increased by 60% for BACE2 (7.01 ± 1.53 in WT+HFD controls vs. 17.63 ± 3.97 in KO+HFD islets, *p* < 0.05) and by 66% for IDE (2.65 ± 1.21 in WT+HFD controls vs. 7.83 ± 1.88 in KO+HFD islets, *p* < 0.05). This increase was specific to BACE2 and IDE, as insulin staining remained comparable between genotypes (Figure 6A–D).

### 3.7. Cav-1 Depletion Reduced TXNIP Expression in iβ-Cav1 KO Mice, and TXNIP Was Found to Co-Localize and Interact with Cav-1

**Figure 7:**
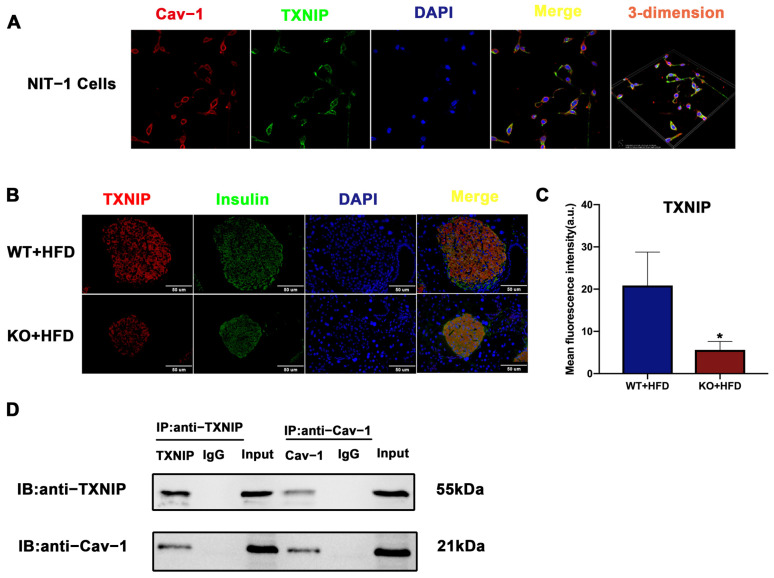
Location and interaction of Cav-1 and TXNIP in NIT-1 cells and iβ-Cav1 KO mice. (**A**): Immunofluorescence results of the Cav-1 (red) and TXNIP (green) in NIT-1cells; (**B**,**C**): Representative fluorescent microscope images (**B**) and quantification of mean fluorescence intensity (**C**) of TXNIP (red) and insulin (green) staining in islets from WT+HFD and KO+HFD mice. Nuclei were counterstained with DAPI (blue). (**D**): Reciprocal co-immunoprecipitation demonstrating physical interaction between Cav-1 and TXNIP in NIT-1 cells. Lysates were immunoprecipitated with anti-TXNIP or anti-Cav-1 antibodies, followed by immunoblotting with the indicated antibodies. IgG served as a negative control. Data here are representative of three independent experiments (n = 3, mean ± SD). * = *p* < 0.05 between KO+HFD group and WT+HFD group. Images of the NIT-1 cells and islets were taken using a microscope under 400× objective lens. Scale bar, 50 μm.

Quantitative co-localization analysis of Cav-1 and TXNIP in NIT-1 cells revealed a strong and consistent spatial correlation, with a mean Pearson’s correlation coefficient of 0.81 ± 0.02 (n = 3) using ImageJ Coloc 2, indicating robust co-localization between the two proteins (Figure 7A). Furthermore, in pancreatic sections, TXNIP fluorescence within insulin-positive β-cells was markedly diminished in KO+HFD mice compared with WT+HFD controls, showing a 73% decrease in mean fluorescence intensity per islet β cell (20.86 ± 7.92 vs. 5.63 ± 1.98, *p* < 0.05; Figure 7B,C). Furthermore, reciprocal co-immunoprecipitation (Co-IP) assays demonstrated that Cav-1 and TXNIP physically interact in NIT-1 cells. Immunoprecipitation of TXNIP pulled down Cav-1, and conversely, immunoprecipitation of Cav-1 pulled down TXNIP, whereas no signal was detected in the IgG control lanes (Figure 7D).

## 4. Discussion

Numerous studies have demonstrated that glucolipotoxicity contributes to the onset and progression of type 2 diabetes mellitus (T2DM) by reducing β-cell mass and impairing β-cell function [2,16]. The underlying mechanisms primarily involve glucose and lipid toxicity, oxidative stress, endoplasmic reticulum (ER) stress, dysregulated autophagy, and islet amyloid deposition derived from human islet amyloid polypeptide (hIAPP) [17]. Reducing the formation of toxic hIAPP oligomers is a promising therapeutic target for type 2 diabetes mellitus (T2DM) treatment. Postmortem studies have detected hIAPP amyloid fibrils in over 90% of examined T2DM patients [18], with the extent of hIAPP deposition negatively correlating with β-cell mass and positively correlating with disease severity [19]. In vitro studies have further confirmed the cytotoxic effects of hIAPP oligomers on β-cells [20]. Moreover, abnormal aggregation of hIAPP is considered to directly contribute to β-cell apoptosis and dysfunction in T2DM [17]. Hence, strategies aimed at reducing hIAPP synthesis and aggregation, such as inhibiting hIAPP production or preventing its aggregation, warrant further investigation.

Caveolin-1 (Cav-1), a principal scaffold protein (21–24 kDa) of caveolae, is highly expressed in pancreatic β-cells and participates in insulin receptor-mediated signal transduction [21]. Our group has previously demonstrated that Cav-1 deficiency protects pancreatic β-cells against palmitate-induced dysfunction and apoptosis. In vitro, Cav-1 silencing in NIT-1 cells and isolated murine islets enhanced glucose-stimulated insulin secretion, reduced apoptosis, and increased cell viability, with mechanistic involvement of attenuated endoplasmic reticulum stress and inflammation [11]. In vivo, using the iβ-Cav1 KO mouse model, we further demonstrated that Cav-1 depletion improved oral glucose tolerance, prevented high-fat-diet-induced hyperglycemia and insulin resistance, and attenuated β-cell apoptosis and inflammation under lipotoxic conditions [11,13]. Other studies have reported a positive correlation between IAPP mRNA levels and islet amyloid deposition in type 2 diabetic patients [22], suggesting that transcriptional upregulation initiates the accumulation of toxic proteins. In the present study, Cav-1 silencing reduced both IAPP protein secretion and expression in NIT-1 cells and isolated islets (in vitro), as well as iβ-Cav-1 KO mouse model islets (in vivo). These findings indicate that Cav-1 silencing not only suppresses IAPP protein expression but also attenuates its secretion. Given that excessive IAPP accumulation is associated with β-cell toxicity in T2DM, these findings further suggest that reducing IAPP synthesis and secretion through Cav-1 modulation may represent a protective mechanism against β-cell dysfunction under lipotoxic conditions [23,24].

In our study, Cav-1 deficiency was associated with significantly suppressed IAPP protein expression, concomitant with downregulation of PAM, PC1, and PC2. The biosynthesis of mature IAPP requires sequential modifications by prohormone convertases PC1 and PC2 and peptidylglycine α-amidating monooxygenase (PAM). Existing evidence indicates that the expression levels of PC1 and PC2 influence the cleavage efficiency of proIAPP [25,26], while the amidation activity of PAM contributes to its post-translational folding stability [27]. This suggests a positive correlation between the levels of PC1, PC2, and PAM and the overall expression of IAPP. Consistent with this, our data revealed synchronized decreases in both mRNA and protein levels of PAM, PC1, and PC2 in Cav-1-deficient NIT-1 cells and mouse islets, suggesting that Cav-1 functions act as a positive regulator sustaining the expression of this “biosynthetic enzyme triad.” In contrast to the up-regulation of PC1 and PC2 accompanied by IAPP accumulation observed in islets of obese rhesus monkeys by Campbell S.A et al. [28], Cav-1 deletion was associated with reduced IAPP production by downregulating these key enzymes, thereby potentially limiting the “substrate supply” for amyloid formation. Importantly, we observed that serum IAPP levels remained comparably elevated under both chow diet and HFD conditions in wild-type mice, suggesting that IAPP expression is already near-maximal under basal metabolic conditions. In contrast, Cav-1 knockout significantly reduced serum IAPP levels under both dietary conditions, with a more pronounced reduction observed under HFD conditions. This differential effect suggests that Cav-1 plays a critical role in sustaining IAPP homeostasis, particularly under metabolic stress where the amyloidogenic burden is heightened, thus supporting our rationale for employing the HFD model to investigate Cav-1-mediated regulation of IAPP proteostasis. The clearance of IAPP primarily relies on insulin-degrading enzyme (IDE), which cleaves IAPP monomers, and β-site amyloid precursor protein-cleaving enzyme 2 (BACE2), which preferentially hydrolyzes oligomers [27]. Our results are consistent with this mechanism, showing that Cav-1 deficiency increases the protein levels of both IDE and BACE2, suggesting enhanced IAPP clearance capacity. While direct measurement of IAPP degradation products was not performed in this study, the coordinated upregulation of these key proteases supports a dual mechanism whereby Cav-1 knockdown both reduces IAPP synthesis and facilitates its degradation.

In addition to enzymes directly involved in IAPP synthesis and degradation, previous studies have reported that TXNIP is associated with the expression of IAPP [29]. Furthermore, Jing et al. identified a TXNIP to miR-124a/FoxA2 to IAPP up-regulation axis, highlighting the synergistic role of TXNIP and IAPP in islet inflammation and β-cell apoptosis in type 2 diabetes [30]. In our study, immunofluorescence confocal microscopy revealed that Cav-1 and TXNIP co-localize in NIT-1 cells. Moreover, co-immunoprecipitation experiments demonstrated a direct physical interaction between Cav-1 and TXNIP (Figure 7D), establishing that these proteins form a functional complex in β-cells. Concordantly, Cav-1 deficiency was associated with coordinate down-regulation of TXNIP and IAPP at both the mRNA and protein levels in both NIT-1 cells and mouse islets. These findings support a model in which Cav-1 positively regulates TXNIP expression, thereby maintaining IAPP synthesis. While rescue experiments will be required to conclusively establish TXNIP as the obligatory mediator, the observed interaction provides mechanistic evidence for this regulatory axis. Xiangfu Jiang et al. also demonstrated that Cav-1 regulates cellular metabolism via TXNIP [31]. Interestingly, their study showed that Cav-1 knockdown in L02 hepatocytes and C57BL/6 mouse models enhanced TXNIP expression, promoted NLRP3 inflammasome activation, and exacerbated liver injury. This apparent discrepancy—where Cav-1 knockdown increases TXNIP in hepatocytes but is associated with decreased TXNIP in our β-cell model—may reflect tissue-specific differences in Cav-1 function. We propose that Cav-1 exhibits high plasticity in modulating lipid raft microstructure across different tissues, though this hypothesis is speculative and requires further investigation. In hepatocytes, Meyer et al. [32] demonstrated that Cav-1 directly binds to the TGF-β type I receptor (TβR-I), thereby inhibiting Smad2/3 phosphorylation. The knockout of Cav-1 significantly enhanced the pro-apoptotic effect of TGF-β. Additionally, Fernández-Rojo et al. [33] reported that Cav-1 facilitates the establishment of cell polarity during liver regeneration by acting as a scaffold to promote Cdc42 guanosine triphosphate (GTP) loading. In contrast, in pancreatic β-cells, Bae et al. [34] found that treatment with high glucose and palmitate up-regulates Cav-1 expression, increases its colocalization with TβR-II, and enhances Smad2/3 phosphorylation, ultimately activating pro-apoptotic genes. Silencing of Cav-1 reversed this apoptotic process. Meanwhile, Nevins et al. [35] showed that Cav-1 functions as a Cdc42 guanine nucleotide dissociation inhibitor (GDI), preventing the binding of GTP to Cdc42. Overexpression of Cav-1 suppressed Cdc42 activity and promoted insulin receptor recycling and sustained insulin secretion. Therefore, the net effect of the Cav-1–TXNIP interaction may depend on the cellular metabolic baseline, lipid raft composition, and inflammatory micro-environment. Our findings not only suggest the protective role of Cav-1 deficiency in pancreatic islets but also emphasize that tissue-specific effects must be carefully considered in future therapeutic strategies targeting Cav-1.

Several limitations of this study should be considered. Firstly, our findings were derived exclusively from murine models (NIT-1 cells, primary mouse islets, and iβ-Cav-1 KO mice), and validation in human β-cell systems remains to be performed. Secondly, rescue experiments—such as re-expression of wild-type Cav-1 in knockdown cells—will be required to conclusively establish Cav-1 as the causal regulator of this enzymatic network. Employing TXNIP overexpression in Cav-1-deficient β-cells will be critical to establish causality. Finally, both hIAPP transgenic mouse models and human islet studies are needed to investigate how Cav-1 regulates toxic hIAPP oligomer formation and thereby impacts β-cell function in T2DM.

## 5. Conclusions

In summary, this study demonstrates that Cav-1 regulates IAPP proteostasis in β-cells through modulation of processing enzymes and degradation factors. Our findings further reveal a direct physical interaction between Cav-1 and TXNIP and indicate that Cav-1 deficiency is coupled with attenuated TXNIP expression, implicating a functional crosstalk between these proteins in IAPP regulation. Future investigations are warranted to fully delineate the downstream phenotypic consequences and to elucidate the precise regulatory network governing β-cell failure in T2DM. Collectively, these findings establish a mechanistic rationale for investigating Cav-1 as a candidate target to mitigate IAPP-related β-cell dysfunction.

## Data Availability

All data generated or analyzed during this study are included in this published article and its Supplementary Information files.

## Supplementary Materials

The following supporting information can be downloaded at: https://www.mdpi.com/article/10.3390/biomedicines14061344/s1, Figure S1: Flowchart of Animal Experiment Procedures; Table S1: Sequence information of quantitative PCR analysis.

## Abbreviations

The following abbreviations are used in this manuscript:

CAV1	Caveolin1
IAPP	Islet amyloid polypeptide
KO	Knock out
WT	Wild-type
CD	Control diet
HFD	High-fat diet
