# Supplementary material for: Caveolin-1 Modulates Islet Amyloid Polypeptide Expression Through Interaction with TXNIP in Murine Pancreatic β-Cells
Source: Biomedicines. 2026 Jun 15;14(6):1344. doi: 10.3390/biomedicines14061344 (PMC13296394; doi:10.3390/biomedicines14061344)
Supplement: Supplementary file 1 [file biomedicines-14-01344-s001.zip › biomedicines-4294214-supplementary.pdf]

Supplementary materials

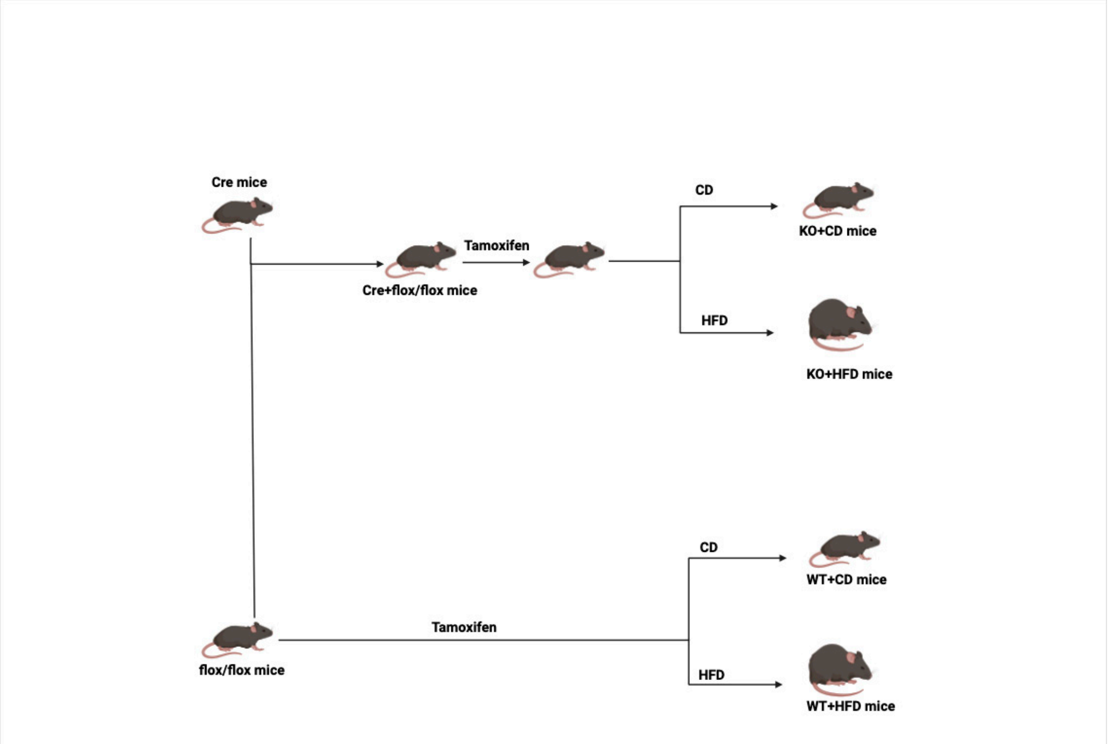

Figure S1. Flowchart of Animal Experiment Procedures

Flox/flox mice were crossed with Cre mice to pruduce Cre+flox/flox mice; Cre+flox/flox mice were administered tamoxifen (i.p., 75 mg/kg body weight for 7 consecutive days) to induce Cre-mediated recombination and achieve β-Cav-1 deletion, insulted in β-Cav-1 knockout mice （KO mice）. All groups, including the WT, and KO groups, were fed either a control diet (CD) (WT + CD group, KO + CD group) or a high-fat diet (HFD) (WT + HFD group, KO + HFD group).

Supplementary Table S1: Sequence information of quantitative PCR analysis

GenBank Accession No.	Gene	Sequence (5'-3')
NM_007616.4	Caveolin-1	Forward: GGGACATCTCTACACTGTTCCCATC<br>Reverse: CTTCTGGTTCTGCAATCACATCTTC
NM_ 010491.3	IAPP	Forward: TGTCGGAAGTGCTACCAAC<br>Reverse: GAGCATTTACACAGGAAGT
NM_ 008878.3	PAM	Forward: CAGAACTATCCCAGAAGAGGC<br>Reverse: TTCTGTTTCTTTGTGATGCCCA
NM_ 013628.3	PC1	Forward: TCTGGTTGTCTGGACCTCTGAGT<br>Reverse: CATCAAGCCTGCCCCATTCTTT
NM_ 008792.3	PC2	Forward: AGCTGGAGAGGTGGATTTTGA<br>Reverse: CTCATCCTGGGAGGACAGA
NM_ 001009935	TXNIP	Forward: TAGCCGGACAAGCTCTGAAA<br>Reverse: AACTCGGGGG CGTACATAAA
NM_ 138681	BACE2	Forward: GAGAGATGAGAATGCCAGTCGCT<br>Reverse: GTAGAAGCCTTCCATCACGGTC
NM_ 013508	IDE	Forward: GGACAGGTTTGCGCAGTTTT<br>Reverse: ACAGCGTTCACCTCTCTGTCTTT
NM_ 007393.5	Actin	Forward: TGTTACCAACTGGGACGACATG<br>Reverse: CTGGATGGCTACGTACATGGCT
